# Respiratory dysbiosis and population-wide temporal dynamics in canine chronic bronchitis and non-inflammatory respiratory disease

**DOI:** 10.1371/journal.pone.0228085

**Published:** 2020-01-28

**Authors:** Aaron C. Ericsson, Alexa R. Personett, Hansjorg Rindt, Megan E. Grobman, Carol R. Reinero

**Affiliations:** 1 College of Veterinary Medicine, University of Missouri, Columbia, Missouri, United States of America; 2 University of Missouri Metagenomics Center, University of Missouri, Columbia, Missouri, United States of America; 3 Department of Veterinary Pathobiology, College of Veterinary Medicine, University of Missouri, Columbia, Missouri, United States of America; 4 Comparative Internal Medicine Laboratory, University of Missouri, Columbia, Missouri, United States of America; 5 Department of Veterinary Medicine and Surgery, College of Veterinary Medicine, University of Missouri, Columbia, Missouri, United States of America; Defense Threat Reduction Agency, UNITED STATES

## Abstract

The lungs of people and companion animals are now recognized to harbor diverse, low biomass bacterial communities. While these communities are difficult to characterize using culture-based approaches, targeted molecular methods such as 16S rRNA amplicon sequencing can do so using DNA extracted from samples such as bronchoalveolar lavage fluid (BALF). Previous studies identified a surprisingly uniform composition of the microbiota in the lungs of healthy research dogs living in a controlled environment, however there are no reports of the lung microbiota of client-owned dogs. Moreover, compositional changes in the lung microbiota depending on disease status have been reported in people, suggesting that similar events may occur in dogs, a species subject to several respiratory disease mechanisms analogous to those seen in people. To address these knowledge gaps, BALF samples from client-owned dogs presenting to the University of Missouri Veterinary Health Center for respiratory signs between 2014 and 2017 were processed for and subjected to 16S rRNA sequencing. Based on specific diagnostic criteria, dogs were categorized as Chronic Bronchitis (CB, *n* = 53) or non-CB (*n* = 11). Community structure was compared between groups, as well as to historical data from healthy research dogs (*n* = 16) of a uniform breed and environment. The lung microbiota detected in all client-owned dogs was markedly different in composition from that previously detected in research dogs and contained increased relative abundance of multiple canine fecal and environmental bacteria, likely due to aspiration associated with their clinical signs. While inter-sample diversity differed significantly between samples from CB and non-CB dogs, the variability within both groups made it difficult to discern reproducible bacterial classifiers of disease. During subsequent analyses to identify other sources of variability within the data however, population-wide temporal dynamics in community structure were observed, with substantial changes occurring in late 2015 and again in early 2017. A review of regional climate data indicated that the first change occurred during a historically warm and wet period, suggesting that changes in environmental conditions may be associated with changes in the respiratory microbiota in the context of respiratory disease. As the lung microbiota in humans and other animals is believed to result from repetitive micro-aspirations during health and in certain disease states associated with dyspnea and laryngeal dysfunction, these data support the increased colonization of the lower airways during compromised airway function, and the potential for temporal effects due to putative factors such as climate.

## Introduction

Despite their anatomic proximity to the heavily colonized oropharynx, the lungs have historically been considered a sterile organ in disease-free states and the presence of clinically relevant bacteria was determined by their ability to grow from aspirates, airway lavages or tissue samples using culture-based methods. However, a substantial proportion of bacterial species colonizing mammalian hosts are, at present, uncultivable using existing techniques[1]. It may therefore not be surprising that several recent studies applying culture-independent sequencing approaches have detected rich microbial communities residing within the distal respiratory tract of healthy humans[2, 3], dogs[4], cats[5, 6], sheep[7], pigs[8], cattle[9], horses[10], chickens[11], and rodents[12]. These microbial communities are repeatedly seeded via subclinical micro-aspiration of oropharyngeal bacteria[13, 14], and in some situations refined for taxa selectively capable of colonizing the bronchial and alveolar tissues[15]. In an effort to understand the relationship between these communities and host health, studies in people have compared the lung microbiota of healthy individuals to that of individuals diagnosed with a variety of inflammatory airway disorders including chronic obstructive pulmonary disease (COPD), asthma, cystic fibrosis, and bronchiectasis[16–21]. The majority of these studies have identified characteristic changes in community structure of the lung microbiota, raising the question of whether those changes initiate disease, promote progression or disease exacerbation, or are merely a sequel to the primary disease process. If such differences in the composition of the lung microbiota are linked to disease pathology, the microbiota might represent novel diagnostic samples, therapeutic targets, or both.

Like humans, dogs are affected with several common inflammatory airway conditions including chronic bronchitis. Chronic bronchitis (CB) in dogs is a non-infectious, inflammatory disorder of the lower airways, and, as in humans, disease is often classified according to the predominant leukocyte population observed in bronchoalveolar lavage fluid[22, 23]. The typical syndrome in dogs is defined by a daily cough greater than 2 months in duration in association with neutrophilic and/or eosinophilic airway inflammation, mucus hypersecretion, and loss of ciliated epithelial cells. Resultant airway caliber changes, ineffective mucociliary clearance, altered lung mechanics, and irreversible architectural changes within the lung are reminiscent of certain forms of human COPD[24]. In dogs, CB is a self-perpetuating, progressive disease frequently without identifiable cause or cure[22]. The current standard of treatment of CB involves lifelong use of corticosteroids to suppress airway inflammation and alleviate clinical signs[23]. Thus, new insights into the pathogenesis of CB are critical to our understanding of the disease and development of novel targeted therapies. Moreover, a better understanding of the influence of the lung microbiota on susceptibility to inflammatory airway conditions in dogs, which often share many of the same environmental pressures as humans, may yield information translatable to similar human conditions.

To evaluate the characteristics of the lung microbiota in dogs diagnosed with CB, bronchoalveolar lavage fluid (BALF) was collected using methods designed to avoid contamination from the upper airways and oropharynx, and subjected to 16S rRNA amplicon sequencing. The richness and composition of these samples were compared to those from two populations of control dogs. The first control group consisted of client-owned dogs with cytologically normal BALF (i.e., lacking infectious organisms, inflammation or neoplastic cells) having cough, labored respiration, stertor (an inspiratory snoring noise created by airflow obstruction between the nares through the nasopharynx), stridor (an abnormal inspiratory noise sometimes described as high-pitched or musical created by airflow obstruction between the larynx and extrathoracic trachea), and/or nasal discharge. As these were client-owned dogs, collection of BALF was deemed medically necessary by the attending clinician’s discretion to evaluate respiratory signs. The risks associated with anesthesia and collection of BALF are minimal but nonetheless present, making it challenging to ethically utilize healthy, client-owned dogs as controls for this study. The second control group was purpose-bred beagles housed in a research setting (historical data[4]). Certain subsets of samples were also interrogated for associations between the lung microbiota (LM) and patient characteristics (e.g., age, body weight, breed), clinical presentation, and seasonality.

## Results

### Patient summary and data quality control

The mean (± SD) age, body weight, and results of BALF cytological examination of the 53 dogs in the CB group and 11 dogs in the non-CB control group are provided in **Table 1**. There were 29 female (all spayed) and 24 male (13 neutered) dogs in the CB group and 10 male (1 neutered) and 1 female (spayed) dogs in the non-CB group. Cough was the predominant clinical sign present in all 53 dogs in the CB group and 9 dogs in the non-CB group. Dogs without CB also had labored respiration (*n* = 3), stertor (*n* = 2), stridor (*n* = 1) and nasal discharge (*n* = 1) with some dogs having more than one clinical sign. The final diagnoses for the 11 dogs without CB included tracheal, mainstem bronchial and/or lobar bronchial collapse (*n* = 4), brachycephalic airway obstructive syndrome (*n* = 2), laryngeal paralysis (*n* = 1), pulmonary osteomas (*n* = 1), tonsillar neoplasia (*n* = 1), and chronic rhinitis (*n* = 1). In 1 dog, a definitive diagnosis was not achieved but there was confirmed absence of lower respiratory tract inflammation.

**Table 1:** Patient demographics in subjects diagnosed with chronic bronchitis (CB) or non-inflammatory respiratory disease (non-CB). SF = spayed female, CM = castrated male, and IM = intact male; BALF = bronchoalveolar lavage fluid.

	CB (*n* = 53)	Non-CB (*n* = 11)
Mean age (±SD)	8 (±4) years	9 (±3) years
Mean weight (±SD)	23 (±19) kg	23 (±16) kg
M/F and status*	29 SF, 13 CM, 11 IM	1 SF, 1 CM, 9 IM
Mean % neutrophils (±SD) BALF	23 (±19) %	2 (±2) %
Mean % eosinophils (±SD) BALF	10 (±12) %	1 (±2) %

Sequencing of DNA recovered from BALF had a mean (± SEM) of 10647 (± 1794) and 12610 (± 3253) reads from samples associated with CB and non-CB respectively. Previously sequenced samples collected from healthy purpose-bred research dogs (*n* = 16) yielded 6314 (± 964) reads and saline flushed through endoscopes (*n* = 3) prior to use yielded 519 (± 179) reads. While there was no significant difference between groups in the number of sequences per sample (*p* = 0.47, Kruskal-Wallis ANOVA on ranks), there was a considerable range in the coverage of samples from dogs with CB or other respiratory disease (**S1 Fig**). While this range overlapped slightly with the read counts obtained from saline rinsed through the bronchoscopes (2 CB samples were below the highest read count obtained from a scope), we opted to leave those two clinical samples in the study due to their strong resemblance to other clinical samples with much better coverage.

### Wide range of lung microbiota compositions in context of airway disease

**Fig 1:**
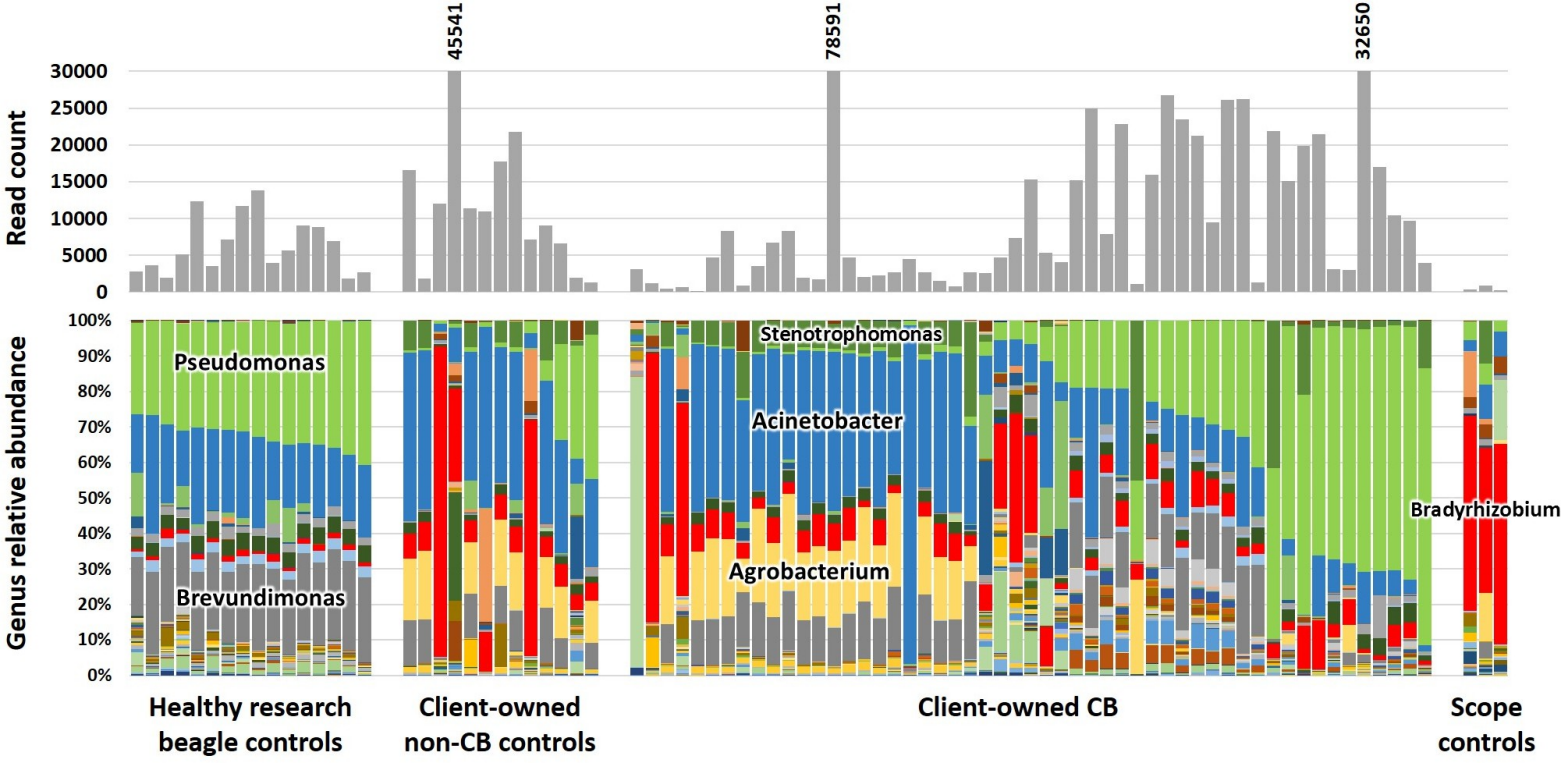
Read counts. Standard (upper) and stacked (lower) bar charts showing read counts and taxonomic relative abundance, respectively, of bronchoalveolar lavage fluid (BALF) from healthy research dogs (*n* = 16), dogs diagnosed with non-bronchitic chronic respiratory disease (non-CB, *n* = 13 samples from 11 dogs), dogs diagnosed with chronic bronchitis (CB, *n* = 53), or sterile water flushed through one of three endoscopes used to collect BALF.

Community profiles at the taxonomic level of genus revealed a similarly broad range of compositions in client-owned dogs affected with inflammatory airway disease. While the BAL of healthy research dogs living in a controlled environment showed a high degree of compositional uniformity dominated by three genera (*Pseudomonas*, *Acinetobacter*, and *Brevundimonas*), samples from client-owned dogs affected with respiratory disease also harbored occasionally high proportions of *Agrobacterium*, *Stenotrophomonas*, *Bradyrhizobium*, (**Fig 1**) and, at lower relative abundance but high prevalence, common canine fecal taxa such as *Fusobacterium*, *Bacteroides*, and *Prevotella* spp. (**S1 Table**). An interesting pattern was noted when comparing read counts to compositional profiles, with seemingly higher read counts associated with *Pseudomonas*-dominated samples from CB dogs. Notably, when including samples from all three dog groups, there was a significant correlation between read count and relative abundance of *Pseudomonadaceae* (**S2 Fig**, R^2^ = 0.28, *p* = 0.01; Spearman’s rank order correlation). When the same analysis was performed in only the CB dogs, the correlation coefficient climbed to 0.546 (*p* < 0.0001), suggesting that an increase in relative abundance of this family is associated with an increase in absolute microbial biomass in the BAL, particularly in the context of CB.

### Airway disease associated with reduced richness and α-diversity

**Fig 2:**
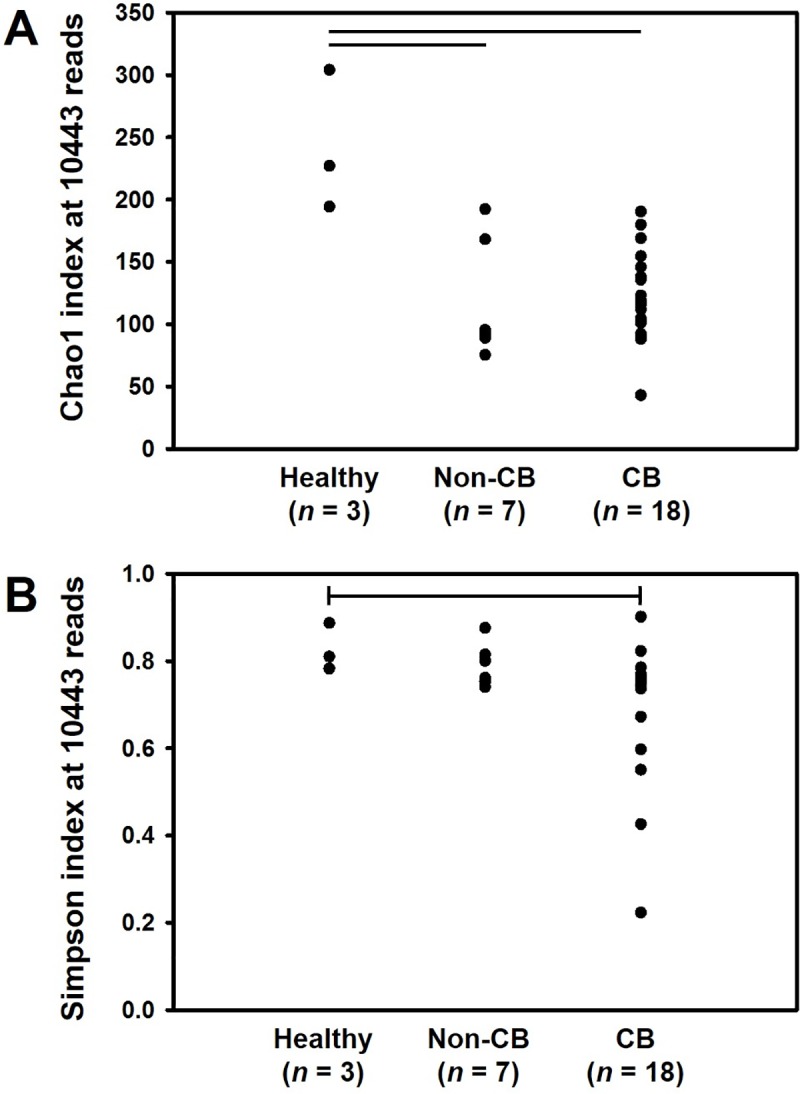
Dot plots showing the Chao1 (**A**) and Simpson (**B**) indices, calculated using a uniform number of randomly subsampled reads (10443/sample), of bronchoalveolar lavage fluid from healthy research dogs (*n* = 3), dogs diagnosed with non-bronchitic chronic respiratory disease (non-CB, *n* = 7), and dogs diagnosed with chronic bronchitis (CB, *n* = 18). Significant overall and pairwise differences (*p* = 0.001) in **A** detected via one-way ANOVA; significant overall difference in **B** detected via Kruskal-Wallis ANOVA on ranks.

Comparison of the predicted true richness (i.e., the number of distinct OTUs detected in a sample) between groups was complicated by the wide range of sequence depths achieved for each sample. Subsampling data to a uniform coverage of 10443 reads per sample allowed for comparison of 3, 7, and 18 samples from healthy research dogs, non-CB dogs, and CB dogs, respectively. One-way ANOVA indicated that samples from both non-CB and CB dogs had a significantly lower richness (i.e., Chao1 index) than samples from healthy research dogs (**Fig 2A**, *p* < 0.001 in both pairwise comparisons). Using the complementary Simpson index of α-diversity (a univariate metric reflects richness as well as how evenly OTUs are distributed within samples), an overall significant difference was detected between the groups, but no differences were detected in *post hoc* pairwise comparisons (**Fig 2B**, *p* = 0.029). While these data suggest that the richness of the lung microbiome is non-specifically reduced in chronic respiratory disease, most likely due to an overgrowth of certain taxa in the setting of inflammation, a less likely alternative interpretation is that client-owned dogs with diverse genetics and environmental conditions, whether affected with airway inflammation or not, have lower richness than inbred beagles housed in a research environment.

### Primary determinant of β-diversity in setting of airway disease is date of collection

**Fig 3:**
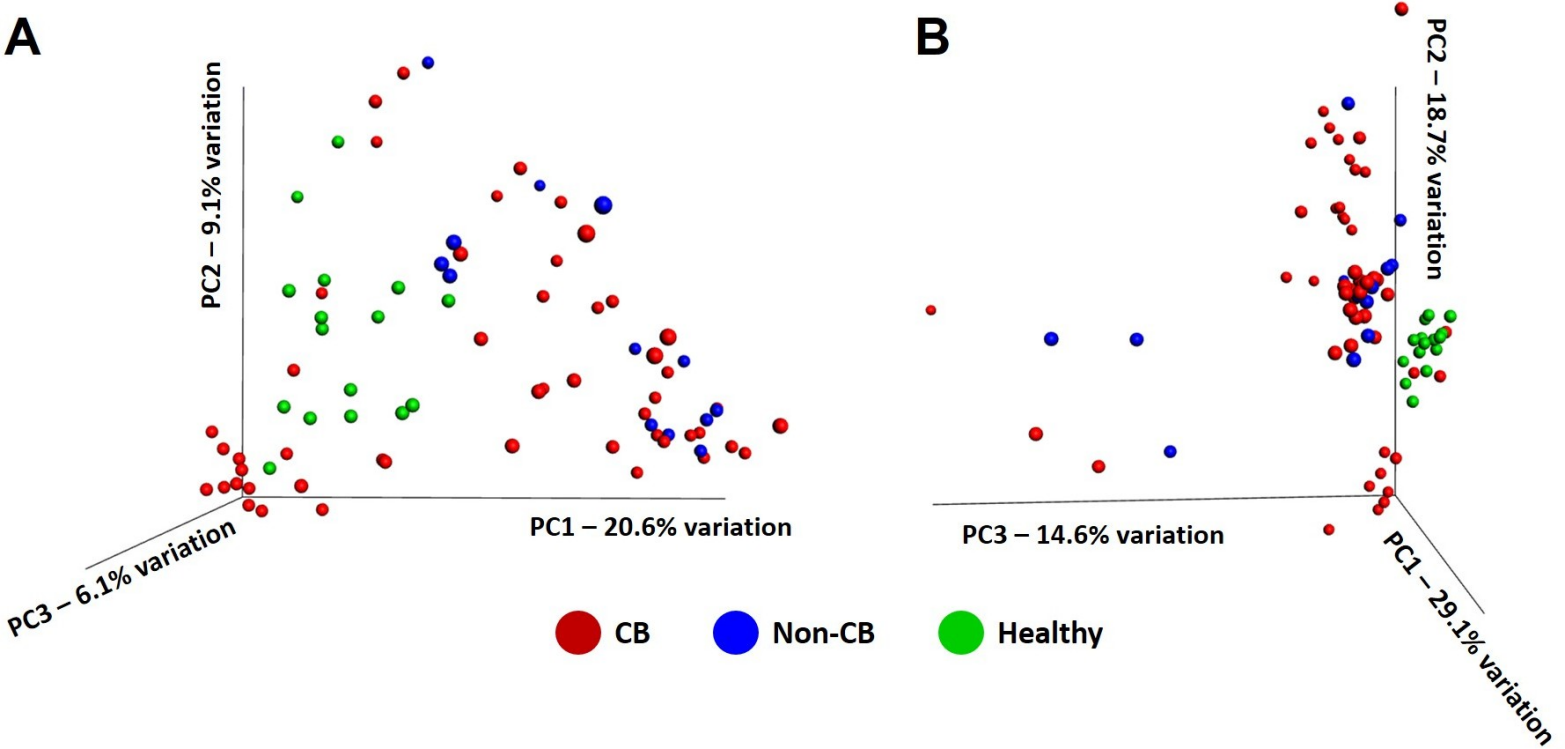
Principal coordinate analysis generated using a uniform number of randomly subsampled reads (1068/sample) and either unweighted (**A**) or weighted (**B**) UniFrac distances, showing the similarity of the microbial communities detected in bronchoalveolar lavage fluid from healthy research dogs (*n* = 16), dogs diagnosed with non-bronchitic chronic respiratory disease (non-CB, *n* = 13 samples from 11 dogs), and dogs diagnosed with chronic bronchitis (CB, *n* = 53).

To visualize similarities or differences in community composition (i.e., β-diversity) between samples, principal coordinate analysis was performed, using both unweighted and weighted UniFrac distances (**Fig 3**). Regardless of the similarity metric used, there was a much greater range of community profiles within the samples from dogs diagnosed with CB relative to that in healthy research dogs or dogs diagnosed with non-CB inflammatory airway disease. While there was absolutely no overlap between samples from healthy and non-CB dogs on ordination of the first three principal coordinates, both of those groups were, at least partially, overlapped by samples collected from CB dogs. PERMANOVA testing of both unweighted and weighted similarities detected significant overall differences between groups (**Table 2**). Pairwise comparisons detected significant differences between samples from healthy dogs and either of the disease groups, whereas differences between samples from CB and non-CB dogs were much less robust or absent, depending on the similarity metric used.

**Table 2:** Results of one-factor permutational multivariate analysis of variance (PERMANOVA) of microbial communities detected in bronchoalveolar lavage fluid collected from healthy research dogs (n = 16), client-owned dogs with chronic bronchitis (CB; n = 48), and client-owned dogs with other chronic airway conditions (n = 13).

Similarity	Overall		Healthy vs. CB		Healthy vs. non-CB		CB vs. non-CB	
	*p*	F	*P*	F	*p*	F	*p*	F
Bray-Curtis	**0.0001**	7.7	**0.0001**	11.5	**0.0001**	23.1	0.13	1.65
Jaccard	**0.0001**	3.4	**0.0001**	4.0	**0.0001**	5.7	0.01	1.91

**Fig 4:**
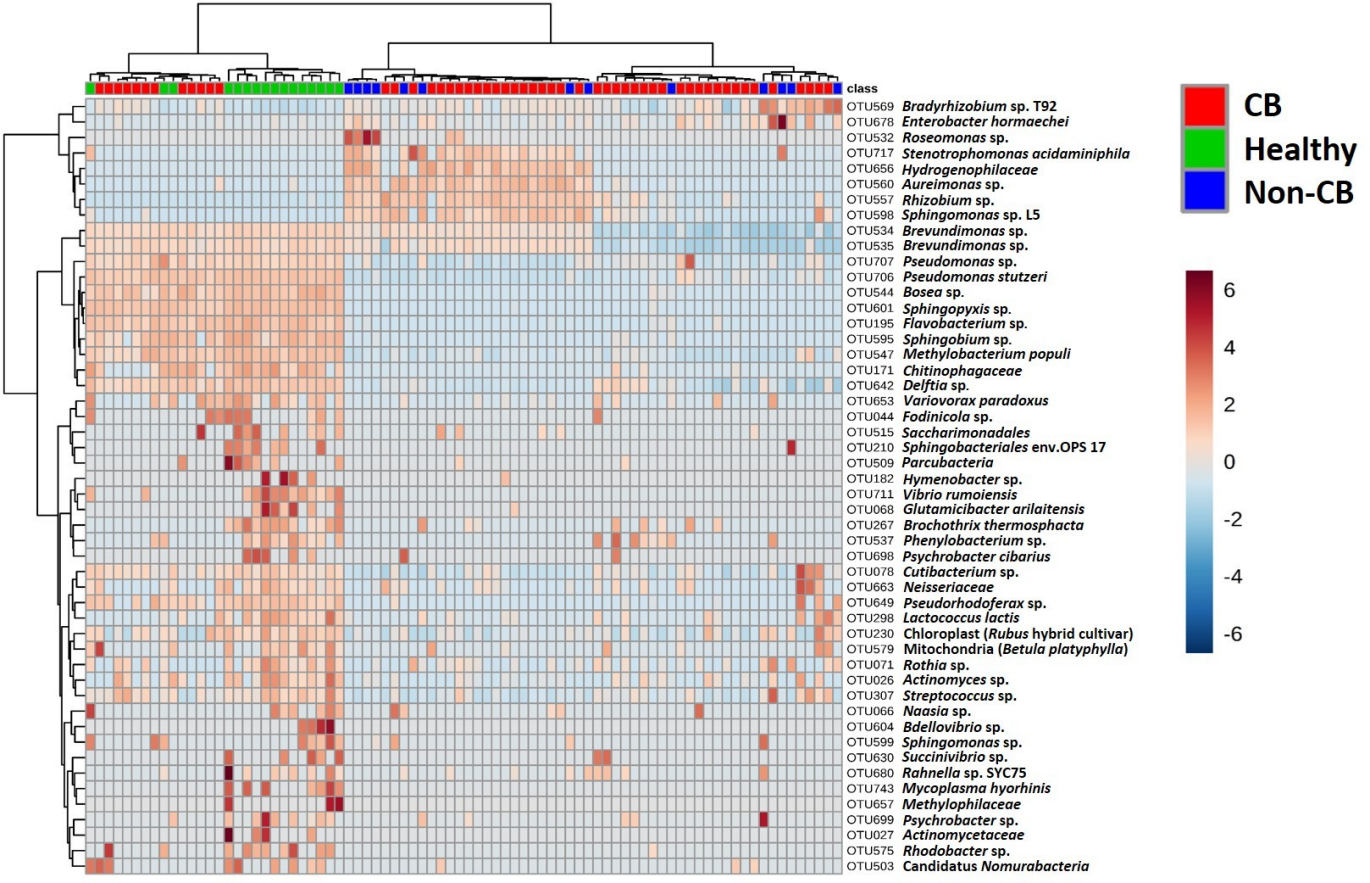
Heatmap showing samples (in columns) and the 50 most variable OTUs between groups (in rows), both clustered using an unweighted pair group method with arithmetic mean (UPGMA) approach. Legend at upper right denotes groups. Color scale (bar at far right) denotes normalized abundance of OTU in a given sample, relative to the abundance in other samples. Best annotation of each OTU is shown at right.

Collectively, these data were interpreted as evidence that the global lung microbiome of dogs affected with CB is highly variable and may not be useful as a classifier of disease status, presumably dependent on other factors such as breed, age, primary etiology, environment, and degree of impairment of mucociliary clearance among others. However, host-associated microbial communities such as the lung microbiota are likely redundant (or random) and changes in select portions of the microbiota associated with inflammation may be obscured by those taxa present at a relatively uniform, or randomly variable (between groups) abundance. Thus, to better detect a ‘signal through the noise’ and identify the primary sources of that signal, hierarchical clustering analysis was performed using only the 50 most variable (between groups) operational taxonomic units (OTUs). Reflecting the PCoA plots, the dendrogram was divided into two primary branches, one of which comprised all of the samples from healthy research dogs and roughly one quarter of the CB dogs, the other comprising all of the non-CB disease dogs and the remainder of the CB dogs (**Fig 4**). The cluster containing samples from healthy dogs and a minority of the dogs diagnosed with CB showed an overall greater relative abundance of the majority of these most variable OTUs, this being most evident in samples from healthy dogs. Specifically, this branch of the dendrogram comprised greater relative abundance of *Brevundimonas* and *Pseudomonas* spp., as well as a number of other Gram-negative *Alphaproteobacteria* with a predominance of ‘environmental’ taxa expressing glycosphingolipids rather than LPS (e.g., *Sphingopyxis*, *Sphingobium*, and *Sphingomonas* spp.) In contrast, the larger cluster containing samples from the non-CB dogs and the majority of CB dogs showed a lower relative abundance of those OTUs, offset by a greater relative abundance of a small subset of OTUs including other glycosphingolipid-expressing microbes (e.g., *Bradyrhizobium*, *Rhizobium*, and *Sphingomonas* spp.) as well as opportunistic pathogens such as *Enterobacter hormaechei*, *Roseomonas*, and *Stenotrophomonas* spp. Overall, those taxa contributing the greatest amount of between-group variability were predominantly Gram-negative *Proteobacteria* although certain Gram-positive *Firmicutes* (e.g., *Streptococcus* sp. and *Lactococcus lactis*) were also present due to their greater abundance in samples from healthy dogs.

**Fig 5:**
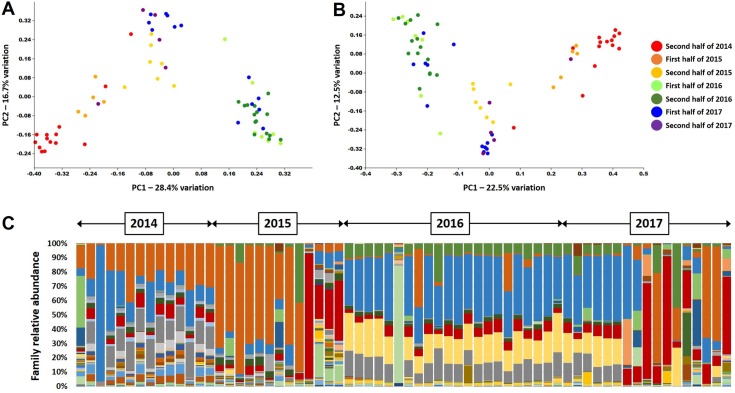
Principal coordinate analysis generated using either Jaccard (**A**) or Bray-Curtis (**B**) similarities, showing similarity of the microbial communities detected in bronchoalveolar lavage fluid from dogs diagnosed with chronic respiratory disease (both CB and non-CB, n = 66), colored according to the 6-month period in which they were collected (legend at right). (**C**) Stacked bar chart showing the relative abundance of families detected in those samples, in temporal order; double-headed arrows above chart denote years.

Attempts to identify other potential sources of variability seen within the CB and non-CB samples, including dog breed, body or skull conformation, age, sex and breeding status, and co-morbidities (e.g., tracheal collapse, brachycephalic airway syndrome, etc.), were largely unsuccessful. However, when samples were stratified by broad time-spans (i.e., 6-month periods) during which the samples were collected, a compelling pattern emerged. Specifically, sample composition was relatively conserved over shorter periods of time and contiguous periods of time were adjacent to each other upon ordination (**Fig 5A and 5B**). As there was no variation in our methods of sample acquisition and processing throughout the entire study, this was interpreted as evidence of a widespread and shared environmental factor contributing to the observed shift in community structure during 2016 and the first half of 2017 (**Fig 5C**).

### Dysbiosis occurring in canine airway disease assumes limited number of dominant structures

**Fig 6:**
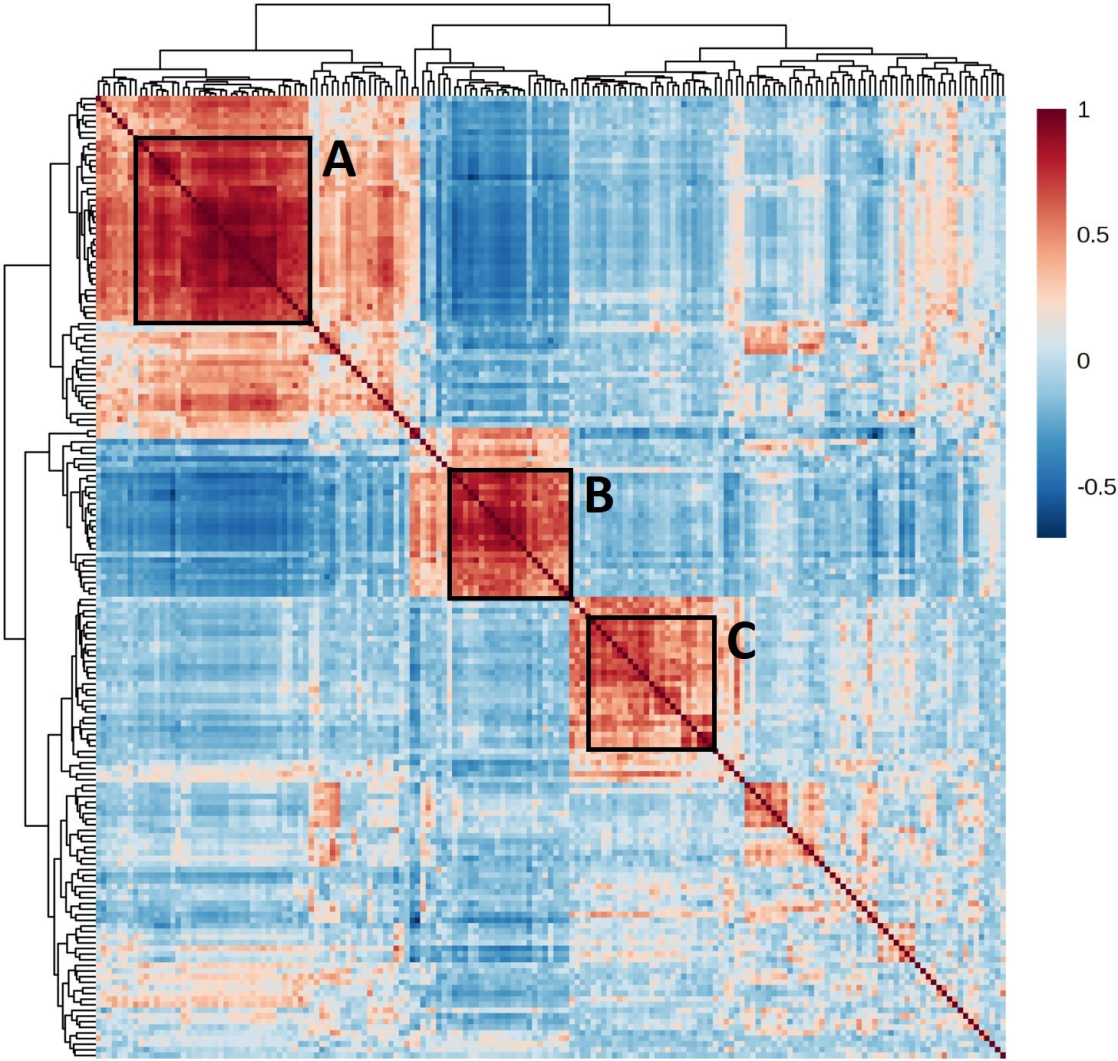
Correlation analysis between cube root-transformed relative abundance of all OTUs detected in bronchoalveolar lavage fluid from dogs diagnosed with chronic respiratory disease (both CB and non-CB, n = 66). Boxed areas represent clusters of highly correlative, and somewhat mutually exclusive, groups of bacteria detected in these samples.

Despite the spectrum of community compositions detected in these samples, they tended to assume somewhat conserved compositions within the sampled population over certain periods of time. To gain a better understanding of the possible syntrophies or cross-feeding networks occurring in the lungs of dogs with chronic respiratory signs, a correlation analysis was performed using data generated from all client-owned dogs (i.e., both CB and non-CB dogs), and filtering for OTUs detected in >20% of samples. OTUs clustered distinctly into one dominant group of positively correlated OTUs and two smaller groups of highly correlated OTUs, referred to here as clusters A, B, and C, respectively (**Fig 6**). These clusters also appeared to be somewhat mutually exclusive, particularly the OTUs present in clusters A and B, and the taxa identified within each cluster are often closely related (**S2 Table**). Specifically, cluster A contained several microbes within the phyla *Bacteroidetes* (*Bacteroides*, *Prevotella*, *Alloprevotella*, and *Flavobacterium* spp.), *Fusobacteria* (three OTUs), and *Firmicutes* (multiple *Ruminococcus*, *Roseburia*, *Blautia*, *Faecalibacterium*, *Allobaculum*, and *Lactobacillus* spp.) that are commonly found in canine fecal samples, along with multiple *Negativicutes* (*Phascolarctobacterium*, *Veillonella*, and *Megamonas* spp.), *Alphaproteobacteria* (*Methylobacterium populi*, *Sphingobium*, *Sphingopyxis*, and *Bosea* spp.), and *Gammaproteobacteria* (*Helicobacter*, *Anaerobiospirilllum*, and *Pseudomonas* spp.). In contrast, while cluster B also contained members of *Bacteroidetes*, *Firmicutes*, *Alphaproteobacteria*, and *Gammaproteobacteria*, few were common fecal commensals and no *Fusobacteria* (a dominant phylum in canine feces[25]) were associated with this cluster. Cluster C also contained a limited number of *Bacteroidetes* and *Firmicutes*, none of which are commonly found in canine fecal samples; several *Gammaproteobacteria* including *Moraxella*, *Campylobacter*, and members of *Neisseriaceae* (*Neisseria* and *Conchiformibius* spp.) and *Pasteurellaceae* (*Pasteurella* and *Frederiksenia* spp.); and *Mycoplasma*. Notably, these *Gammaproteobacteria* (and *Mycoplasma*) are dominant taxa detected in oropharyngeal swabs of healthy dogs[4]. Interestingly, when the relative abundance and prevalence of the OTUs found in clusters A, B, and C were compared against the year in which the sample was collected, a clear pattern emerged with the co-segregating microbes in cluster A being, collectively, more prevalent across the population and present at a higher relative abundance in samples collected in 2014, while the microbes co-segregating in cluster B were, collectively, more prevalent and at a higher relative abundance in samples collected in 2016. Notably, the microbes found in cluster C were frequently most prevalent and at greatest mean relative abundance across the population in samples collected in 2017.

Collectively, these data suggest the occurrence of relatively conserved constellations of microbes within the lungs of client-owned dogs affected with chronic respiratory signs. As these bacterial clusters appear to change in composition across the population over time, and do not show clear correlations to clinical presentation, we speculate that, regardless of the primary defect, the compromised airways of these dogs allows colonization of the lungs by temporally dynamic communities present in the environment. These findings support a microbial connection between host and environment and introduce climate and regional geography into considerations of precision medicine.

## Discussion

Overall, this study documented larger variability in coverage and decreased richness in client-owned dogs with inflammatory airway disease (CB) and non-inflammatory respiratory disease (non-CB) when compared to healthy research dogs in a controlled environment. Respiratory dysbiosis, defined as deviation from a healthy respiratory microbiome, has been documented in many airway and parenchymal disorders in humans and this study similarly describes dysbiosis in pet dogs with CB and non-CB respiratory disease. Microbial community composition among BALF samples, overall and when focusing on the most discriminatory OTUs, was most variable within the group of dogs with CB compared to non-CB and healthy dogs samples. These more pronounced differences in microbial composition in dogs with CB are perhaps reflective of their greater impairment in mucociliary function. Additionally, bacterial communities revealed distinct “themes” over time noted in client-owned dogs with and without CB, raising the strong possibility of environmental contributors to the respiratory microbiome of dogs with impaired mucociliary function.

The composition of microbial communities in the respiratory tract is influenced by microbial immigration (microaspiration, bacterial inhalation, and direct mucosal dispersion), emigration (mucociliary clearance, cough, and innate/adaptive immunity), and relative reproduction rates of microbes[26]. The lung microbiota of dogs shares many of the same genera found in humans. In both species, there appears to be a regional continuity between the bacterial communities present in the oral cavity, upper airways, and lung[2–4]. It is speculated that the lower airways are seeded in a somewhat stochastic manner via repeated ‘silent’ microaspiration, occurring in roughly 50% of healthy adults during sleep[13]. Clinically significant repetitive microaspiration has been identified in a variety of respiratory disorders including, but not limited to, COPD in humans[27] and CB in dogs[28, 29]. Compared to immigration of bacteria, we expected emigration to be more substantially impacted in the client-owned dogs with CB compared to those with other types of non-inflammatory airway disorder. Mucociliary clearance is the primary defense mechanism of the lung and it relies on appropriate function of ciliated epithelial cells and mucous glands in the lower respiratory tract[30]. Impaired mucociliary clearance in large part due to pathologic mucous hypersecretion is an important feature of inflammatory lower airway disorders[31] and is generally absent or minimal in the other non-inflammatory respiratory diseases. Microbial immigration, emigration, and relative reproductive rates likely help explain the presence of mutually exclusive groups of bacteria suggesting semi-distinct compositional profiles in the majority of dogs with CB and non-CB respiratory disease. Despite the normal BALF cytology, some of the non-CB respiratory disorders (e.g., tracheal collapse, laryngeal paralysis, brachycephalic airway syndrome) may also be associated with increased risk of repetitive microaspiration and defective clearance that could contribute to the noted respiratory dysbiosis compared with healthy research dogs.

It is unclear whether the microbiota found in healthy lungs confers colonization resistance to pathogenic or competing organisms as does the gut microbiota[32]. Rather, we speculate that in dogs with CB and non-CB the decreased richness detected in samples was associated with an increased biomass comprising a limited number of fecal or oropharyngeal commensals, representing samples dominated by microbial clusters A and C (**Fig 6**), respectively. In humans with COPD, BALF samples with transcriptionally-active bacteria including *Pseudomonas* had significantly higher bacterial loads than BALF samples enriched with other bacteria like *Escherichia*[33]. Furthermore, these COPD patients with *Pseudomonas* had a stronger T helper 17 cell immune response and higher rates of disease exacerbation, highlighting the presence of host-microbe interactions[33]. Similarly in our study, dogs with CB had increased relative abundance of *Pseudomonas* with increasing absolute biomass. Future studies investigating the interactions between host immunity and specific bacteria as well as overall community microbial composition in canine CB are warranted.

Regarding the bacteria found in greater proportion in client-owned dogs with respiratory disease relative to previously sampled healthy research dogs (**S1 Table**), there are five taxa of note. The *Alphaproteobacteria Agrobacterium radiobacter* and *Bradyrhizobium* sp. T92, from the families *Rhizobiaceae* and *Bradyrhizobiaceae* respectively, are soil organisms not associated with any mammalian disease processes. Notably, these organisms were also identified in samples from healthy dogs (including *Bradyrhizobium* at a mean relative abundance of 1.4% and 100% prevalence). In contrast, the *Gammaproteobacteria Enterobacter hormaechei* and *E*. *coli*, detected at a higher prevalence and in greater relative abundance in samples from dogs with respiratory disease, are fecal coliforms from the family *Enterobacteriaceae*. Both have been associated with respiratory tract (and other) infections, often in association with hospitalization and mechanical ventilation. Similarly, *Stenotrophomonas maltophilia* (Family *Xanthomonadaceae* within class *Gammaproteobacteria*), detected at comparable prevalence but much higher relative abundance in samples from dogs with respiratory disease when compared to historical data from healthy dogs, is considered an “emerging global opportunistic pathogen” associated with multidrug resistance. Notably, *S*. *maltophilia* is often recovered from polymicrobial respiratory infections in high-risk settings such as individuals diagnosed with cystic fibrosis (along with *P*. *aeruginosa*) and patients receiving mechanical ventilation. Lastly, while certain *Pseudomonas* sp. were detected at significantly greater relative abundance in client-owned dogs with respiratory disease, those same OTUs (i.e., *Pseudomonas* sp. DQ-01 and *P*. *hibiscicola*) were both detected at high prevalence in healthy dogs, alongside a different highly dominant (*P*. *stutzeri*). Collectively, we interpret this as evidence that *Pseudomonas* spp. are normal colonizers of the canine lung in health and disease, and whether the differences between affected and healthy dogs in the dominant strains reflect differences in environmental exposures or the health status of the dogs cannot be determined.

A review of climate data from the region in which the majority of these dogs reside (central Missouri) revealed that, since 1895, 2015 was the 4^th^ wettest year on record, had the 5^th^ warmest Autumn on record, and the warmest December on record[34]. Climate change affects air quality and leads to alterations in microbial (predominantly bacteria and fungi) and non-microbial elements, both with potential to impact respiratory tract health. Along with pollen, spores and plant debris, a substantial proportion of the organic carbon fraction of outdoor airborne particulate matter is contributed by bacteria and fungi[35, 36]. The community composition of bacterial and fungal communities within indoor environments have been shown to be influenced both by outdoor factors as well as passage of time[37]. As supported by the results of this study, time (year) of BAL collection significantly impacted microbial community composition in client-owned dogs. If the observed differences over time in clinical samples are indeed associated to differences in airborne bacterial communities, one would speculate that geographic differences would exert a similar influence. Clearly, additional studies are needed to better understand the influence of environmental factors on the respiratory microbiome of both dogs and people, the degree to which those influences change in health versus disease, and how to interpret host-associated data in the context of the host’s environment. It should be noted that the samples were collected and processed using the same techniques and methods throughout the study duration, sequencing chemistries and primer sets were consistent, and all sequencing data were concatenated, filtered, and annotated in a single analysis. With the exception of two samples, all experimental samples returned read counts between one and two orders of magnitude greater than endoscopic rinse controls, obviating the possibility that this change across time is due to some sort of contamination.

Lastly, we again fully acknowledge the limitations associated with both sets of controls used in the current study. It is extremely challenging to identify a ‘perfect’ control for client-owned dogs diagnosed with CB due to the inherent risks associated with anesthesia and BALF collection. All BALF samples from CB and non-CB dogs were collected as part of the routine diagnostic evaluation and unnecessary collection of such samples from healthy client-owned dogs would be unethical. While we used dogs with normal BALF cytology as one control, these dogs nonetheless presented with respiratory clinical signs and cannot be considered healthy *per se*. In contrast, the samples collected from healthy research dogs provide a better control with regard to health status, however the fact that these are not client-owned dogs presents a different, albeit unavoidable, confounding factor. Owing to these unavoidable confounds, the data described above do not distinguish between the possibility that the high degree of diversity (i.e., β-diversity) seen among the client-owned dogs is a reflection of their health status or their environment. Regardless of those causal relationships, the changes in bacterial community structure over time within the host (i.e., dog) population raise questions about the factors controlling those population-wide changes. Such temporal fluctuations in respiratory tract microbial communities have not been described previously and additional studies are needed, including longitudinal assessments following treatment to better understand the timing of changes in the lung microbiota and the development of clinical signs, as well as the environmental factors influencing the lung microbiota.

In summary, the dominant finding of the present study is that the lung microbiota of dogs affected with chronic respiratory disease is affected by dysbiotic changes that segregate over time with regard to β-diversity. The fact that these disease-associated communities assume a seemingly limited range of dominant characteristic community structures begs the question of differences in the function, metabolism, or influence of these different communities in dogs suffering from CB or non-inflammatory conditions. Recognizing that disease is likely going to be accompanied by some form of lung microbiome ‘dysbiosis’, should one type be preferred? Future studies will rely on increasing sample sizes and careful, comprehensive documentation of environmental and clinical factors at the time of sample collection.

## Materials and methods

### Ethics statement

Study was approved by the University of Missouri Institutional Animal Care and Use Committee (MU IACUC protocol #8240). BALF samples were collected for medically necessary reasons in client-owned dogs brought to the MU Veterinary Health Center (VHC) and prospectively banked for study.

### Animals

Animals enrolled in the study included 53 client-owned dogs diagnosed with spontaneous CB and 11 dogs (13 BALF samples) with respiratory clinical signs in the absence of lower airway inflammation or infection at the MU VHC (non-CB). Patient data were gathered from the medical records system of the MU VHC including signalment, respiratory clinical signs, concurrent medications, comorbidities, and bronchoalveolar lavage fluid (BALF) cytology. Inclusion criteria for the CB group consisted of a history of idiopathic cough > 2 months in duration and evidence of airway inflammation based on BALF cytology. We defined this as >7% non-degenerate neutrophils, >7% eosinophils, or both, based on previously published normal BALF criteria in dogs[38]. Dogs in the non-CB group had a respiratory evaluation at the clinician’s discretion inclusive of BALF collection; unremarkable BALF was defined as having ≤7% non-degenerate neutrophils and ≤7% eosinophils. Exclusion criteria for both groups included dogs treated with antibiotics or corticosteroids within 2 weeks of time of BALF collection. Information regarding the healthy research dogs from which the reference data were generated is available here[4].

### Bronchoalveolar lavage fluid collection and analysis

BALF samples were collected as part of the diagnostic evaluation of dogs with respiratory clinical signs at the MU VHC. Anesthetic protocols, performed at the discretion of a board-certified veterinary anesthesiologist, were tailored to each dog based on hematologic and biochemical analyses, cardiopulmonary status and identified co-morbid conditions. Patients were anesthetized and intubated using a sterile endotracheal tube (for blind BALF collection) or oxygen was provided using a red rubber catheter during direct intubation using a bronchoscope. BALF samples were collected using a single 20 mL aliquot of sterile saline via a blind or endoscope-guided technique. The MU VHC Clinical Pathology Department performed differential cell counts and cytologic examinations on Wright’s-stained cytospin preparations. The percentages of neutrophils and eosinophils were quantified. Aliquots of unused BALF were stored at -80°C until DNA extraction.

### DNA extraction

DNA extraction of BALF samples was performed using the previously described protocol[4]. BALF samples were centrifuged at 5000 × g for 10 minutes to pellet bacterial cells. The supernatant was removed, and the pellet was resuspended in 800 μL of lysis buffer. Samples were incubated at 70ºC for 20 minutes with intermittent vortexing, followed by centrifugation at 5000 × g for 5 minutes. Supernatant was transferred to a 1.5 mL Eppendorf tube, and 200 μL of 10 mM ammonium acetate were added to each sample and briefly mixed. Samples were chilled on ice for 5 minutes and centrifuged at 5000 × g for 15 minutes. The supernatant was transferred to a new Eppendorf tube, and one volume of chilled isopropanol was added. Samples were held on ice for 30 minutes, then centrifuged at 16,000 × g at 4°C for 15 minutes. The DNA pellet was washed with 70% ethanol and resuspended in 150 μL of Tris-EDTA (10 mM Tris and 1 mM EDTA). Using Qiagen DNeasy Blood and Tissue kits, 15 μL of proteinase K and 200 μL of AL Buffer were added to each tube. Samples were incubated at 70°C for 10 minutes, and 200 μL of 100% ethanol was added to the tubes. Once mixed, the samples were transferred to a DNeasy kit spin column, and the DNA was purified according to the manufacturer’s instructions and eluted in 200 μL of EB buffer. Fluorometry (Qubit dsDNA BR assay, Life Technologies, Carlsbad, CA) was used to determine the DNA concentration of each sample. Samples yielding less than 216 ng of DNA were concentrated in a Sorvall SpeedVac centrifuge until approximately that concentration or reaching a minimum volume of 60 μL. Samples were stored at -20°C until PCR and sequencing.

### 16S rRNA Library preparation and sequencing

Library construction and sequencing were performed at the MU DNA Core facility. Bacterial 16S rRNA amplicons were produced by amplification of the V4 hypervariable region of the 16S rRNA gene with single-indexed universal primers (U515F/806R) flanked by Illumina standard adapter sequences[39, 40] and oligonucleotide sequences available at proBase[41]. Dual-indexed primers with unique index sequences were used in all reactions. PCR reactions (50 μL) containing 100 ng of genomic DNA, F and R primers (0.2 μm each), dNTPs (200 μm each), and Phusion High-Fidelity DNA Polymerase (1U) were subjected to the following cycling parameters: 98°C^(3:00)^ + [98°C^(0:15)^ + 50°C^(0:30)^ + 72°C^(0:30)^] × 25 cycles + 72°C^(7:00)^. Amplicon libraries from each pool (5 μL) were pooled, thoroughly mixed, and purified via addition of Axygen AxyPrep MagPCR Clean-up beads to an equal volume of 50 μL and incubated at room temperature for 15 minutes. Products were washed multiple times in 80% ethanol, dried, resuspended in 32.5 μL EB buffer (Qiagen), incubated at room temperature for 2 minutes, and then placed on a magnetic stand for 5 minutes. The final amplicon pool was evaluated using the Advanced Analytical Fragment Analyzer automated electrophoresis system, quantified using the quant-iT HS dsDNA reagent kits (Invitrogen) with a Qubit 2.0 fluorometer, and diluted according to the manufacturer’s protocol for sequencing on the Illumina MiSeq platform with 2×250 bp paired-end reads.

### Informatics processing

Assembly, binning, and annotation of contiguous sequences of DNA were performed at the MU Informatics Research Core Facility. Contiguous sequences (contigs) were assembled using FLASH software[42] and culled if found to be far below the expected length of 292 bp after trimming for a base quality of less than 31. Qiime v1.9 software[43] was used to perform *de novo* and reference-based chimera detection and removal, and remaining contigs were assigned to operational taxonomic units (OTUs) using a criterion of 97% nucleotide identity. Briefly, cutadapt[44] (https://github.com/marcelm/cutadapt) was used to remove the primers at both ends of the contig and reject contigs that did not contain both primers. The usearch[45] fastq_filter (http://drive5.com/usearch/manual/cmd_fastq_filter.html) command was used for quality trimming of contigs. Contigs were rejected if the expected number of errors was greater than 0.5. All contigs were clipped to 248 bases and shorter contigs were rejected. The Qiime command split_libraries_fastq.py is used to clean and assemble contigs with the outputs for all samples concatenated into 1 seqs.fna file for clustering by the uparse method (http://www.drive5.com/uparse/). The uparse[46] method was used to both cluster contigs and remove chimeras. Taxonomy was assigned to selected OTUs using BLAST[47] against the SILVA database v128[48] of 16S rRNA sequences and taxonomy.

### Statistical analysis and multivariate comparisons

Univariate data (e.g., read counts, richness or α-diversity of individual samples) were first tested for normality using the Shapiro-Wilk method, and equal variance using the Brown-Forsyth method. Normally and non-normally distributed data were then tested for differences using one way analysis of variance (ANOVA) or Kruskal-Wallis one-way ANOVA on ranks, respectively. Post hoc pairwise comparisons were then performed using the Holm-Sidak or Dunn’s method, respectively. Correlations between univariate data were performed using Spearman’s rank order correlation. Testing described above was performed using SigmaPlot 14.0 (Systat Software). Testing for differences in multivariate data (e.g., β-diversity between groups) was performed using permutational multivariate analysis with 9999 permutations for all analyses. For all such comparisons, both Jaccard (unweighted) and Bray-Curtis (weighted) similarities were used. PERMANOVA and principal coordinate analyses were performed using Past 3.22[49]. Hierarchical clustering using an unweighted pair group method with arithmetic mean (UPGMA) and correlation analysis of OTU relative abundance were performed using MetaboAnalyst software[50].

## Supporting information

S1 Fig Number of high-quality reads obtained from bronchoalveolar lavage fluid (BALF) of healthy research dogs (*n* = 16), dogs diagnosed with non-bronchitic chronic respiratory disease (non-CB, *n* = 13 samples), dogs diagnosed with chronic bronchitis (CB, *n* = 53 samples), or sterile water flushed through one of three endoscopes used to collect BALF. No significant differences were detected between treatment groups (*p* = 0.47, Kruskal-Wallis ANOVA on ranks).
(JPG)

S2 Fig Dot plot showing correlation between the number of reads obtained per sample and the detected relative abundance of bacteria within the family *Pseudomonadaceae* (R^2^ = 0.28, *p* = 0.01, Spearman’s rank order correlation). (JPG)

S1 Table The mean (and standard deviation) relative abundance, and percent incidence, of all operational taxonomic units detected at a mean relative abundance of 0.5% in at least one group. (XLSX)

S2 Table Taxonomy of Operational Taxonomic Units (OTUs) found in three co-segregating clusters of bacteria (see Fig 6), detected in the bronchoalveolar lavage fluid collected from dogs affected with chronic bronchitis (CB; *n* = 53 samples) or non-bronchitic chronic respiratory disease (non-CB; *n* = 13 samples); last two columns denote the year demonstrating the greater relative abundance (RA) or overall prevalence, respectively, of each OTU. (XLSX)
