# Supplementary figures and images for: Respiratory dysbiosis and population-wide temporal dynamics in canine chronic bronchitis and non-inflammatory respiratory disease
Source: PLoS One. 2020 Jan 28;15(1):e0228085. doi: 10.1371/journal.pone.0228085 (PMC6986754; doi:10.1371/journal.pone.0228085)

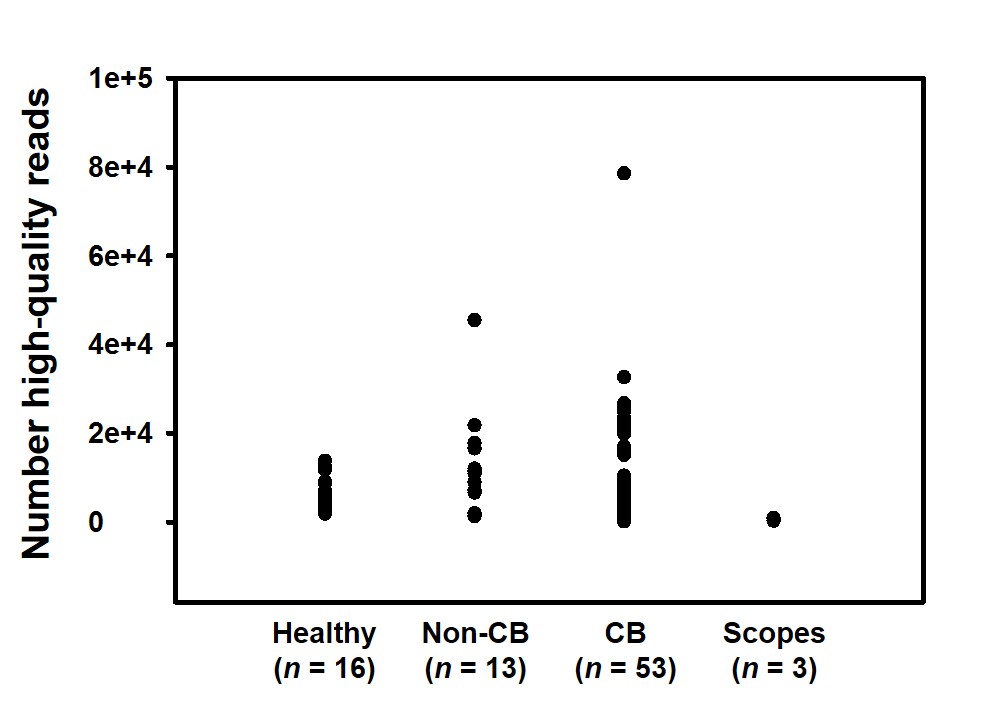

Supplement: S1 Fig — No significant differences were detected between treatment groups (p = 0.47, Kruskal-Wallis ANOVA on ranks). (JPG) [file pone.0228085.s001.jpg]

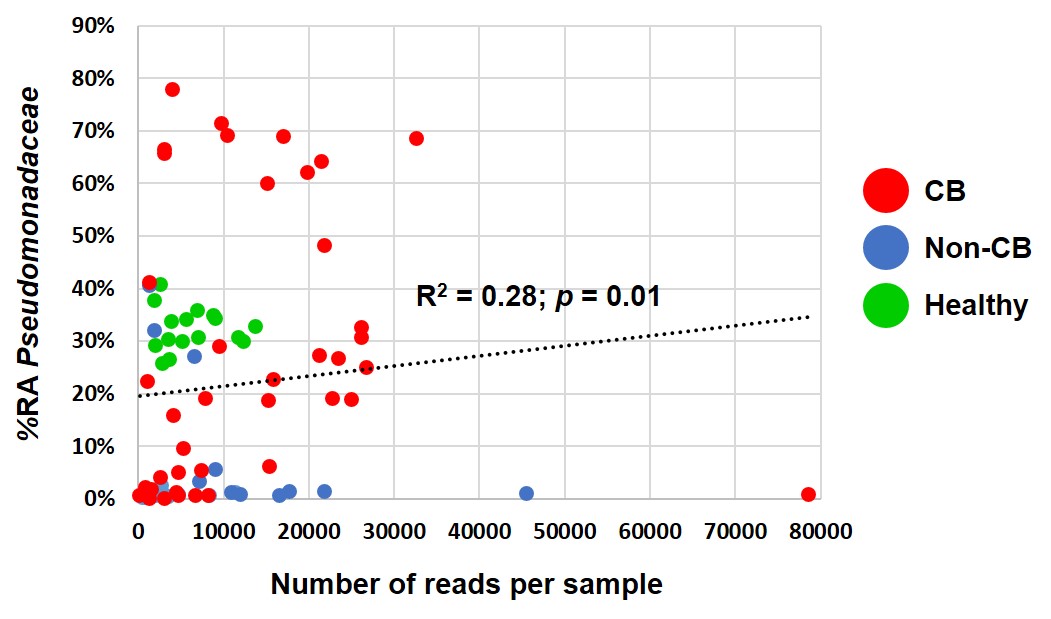

Supplement: S2 Fig — (JPG) [file pone.0228085.s002.jpg]
